# Benzodiazepine and antipsychotic medication use in older adults

**DOI:** 10.1002/nop2.425

**Published:** 2019-11-25

**Authors:** Michael W. Neft, Sarah Oerther, Shannon Halloway, Sandra K. Hanneman, Ann M. Mitchell

**Affiliations:** ^1^ Department of Nurse Anesthesia University of Pittsburgh School of Nursing Pittsburgh Pennsylvania; ^2^ Saint Louis University School of Nursing St. Louis Missouri; ^3^ Department of Community, Systems, and Mental Health Nursing Rush University Chicago Illinois; ^4^ Department of Research Cizik School of Nursing University of Texas Health Science Center at Houston Houston Texas; ^5^ Health and Community Systems Department University of Pittsburgh School of Nursing Pittsburgh Pennsylvania

## 1 INTRODUCTION

The prescription of benzodiazepine and antipsychotic medications has been rising for the past fifteen years for all populations (Bachhuber, Hennessy, Cunningham, & Starrels, 2016; Foster et al., 2016; Olfson, King, & Schoenbaum, 2015; Sohn, Moga, Blumenschein, & Talbert, 2016). In older adults, persons over the age of 65 years, there is an even greater increase in prescriptions of benzodiazepines and antipsychotic medications with increasing age (Foster et al., 2016; Gerlach, Wiechers, & Maust, 2018; Olfson et al., 2015). Additionally, older adult women are prescribed benzodiazepines at nearly two times the rate as older adult men (National Institutes of Health, 2014).

Benzodiazepines and antipsychotic medications are often prescribed off‐label for older adults to manage insomnia, delirium and dementia (Gareri et al., 2014; Kate, Pawar, Parkar, & Sawant, 2015; Markota, Rummans, Bostwick, & Lapid, 2016). For instance, antipsychotic medications were ordered—without a diagnosis for psychosis—for 17% of 19,780 older adults within 100 days of admission to a long‐term care facility and for 24% within one year of admission (Rochon, 2019). Common problems with off‐label prescribing of benzodiazepine and antipsychotic medications for older adults include alterations in pharmacokinetics and pharmacodynamics due to age, and adverse reactions.

## 2 ALTERATIONS IN PHARMACOKINETICS AND PHARMACODYNAMICS

With ageing, there are changes in pharmacokinetics (Porter, Kaplan, Lynn, & Reddy, 2018)—the way drugs move through the body during absorption, distribution, metabolism and excretion—with decreased metabolism and excretion of many benzodiazepine and antipsychotic medications in older adults (Porter et al., 2018; Zubenko, 2000). Toxicity may develop slowly because concentrations of chronically used drugs increase until a steady state is achieved. Certain benzodiazepines (e.g. diazepam, flurazepam) take much longer to be excreted from the body in older adults, compared with younger adults, and signs and symptoms of toxicity may not appear until days or weeks after therapy is started (Porter et al., 2018; Zubenko, 2000).

Likewise, there are changes in pharmacodynamics—the effects of drugs on the body—with increased sensitivity to benzodiazepine and antipsychotic medications in older adults (Porter et al., 2018; Zubenko, 2000). Due to a less efficient blood–brain barrier, the brain is exposed to increased drug levels in the older adult, causing confusion, a higher degree of sedation than desired and impairment of psychomotor performance, contributing to falls and fractures. Antipsychotic medications may result in tardive dyskinesia, which involves abnormal muscle movements in the face, eyes, mouth, tongue and limbs. These symptoms, in some cases, may be irreversible even after the medication has been discontinued (Porter et al., 2018; Zubenko, 2000).

## 3 ADVERSE REACTIONS

The American Geriatrics Society and the Hartford Institute for Geriatric Nursing have strongly advised against off‐label use of benzodiazepines in older adults. This is due to the increased risk for adverse drug reactions such as central nervous system depression, aspiration pneumonia, falls, hospital admission and death. (Markota et al., 2016). Similarly, the American Geriatrics Society and the Hartford Institute for Geriatric Nursing have strongly advised against off‐label use of antipsychotic medications, which increases risk for adverse drug reactions including orthostatic hypotension, cerebrovascular events, sedation, fatigue, extrapyramidal symptoms and cognitive deficits (Gareri et al., 2014; Kate et al., 2015). Additionally, healthcare providers with prescription authority have shared their view that benzodiazepines represent, “our other prescription drug problem,” noting that the total number of off‐label prescriptions dramatically increased over the past decade while prescriptions of benzodiazepines by psychiatrists remained steady (Lembke, Papac, & Humphreys, 2018).

## 4 ALTERNATIVE EVIDENCE‐BASED PRACTICE

Nurses should advocate alternative strategies to off‐label prescription of benzodiazepine and antipsychotic medications in older adults to prevent central nervous system depression, aspiration pneumonia, falls and cognitive deficits (Shaw et al., 2019). Psychotherapy, stress management techniques and cognitive behavioural therapy (CBT) are all alternatives to consider besides prescribing benzodiazepines (Maust, Kales, Wiechers, Blow, & Olfson, 2016).

Additional alternatives to the off‐label prescription of antipsychotic medications include examining the patient's environment for stressors and modifying these stressors accordingly. Any changes in the patient's life should be carefully planned for so that they are executed in the best, safest way possible for the patient. If changes are implemented smoothly, resulting delirium or anxiety may be minimized and medications avoided. Daily living should be structured so that demands or challenges are within manageable limits, and a positive atmosphere is created (Lyketsos et al., 2006). Again, the ultimate goal of appropriately structured living with manageable challenges is avoidance of off‐label prescription of benzodiazepine and antipsychotic medication for the management of insomnia, delirium and dementia.

## 5 CONCLUSION

The negative impacts of benzodiazepines and antipsychotic medications in older adults are partly related to pharmacokinetics and pharmacodynamics in this population and partly related to off‐label prescribing habits. Nurses need to advocate for their patients by raising awareness to alternative evidence‐based practice measures to manage insomnia, delirium and dementia in older adults. Nurses need to educate older adults and their family members about benzodiazepine and antipsychotic medications including indications for use, side effects, and indications for discontinuation for treatment of psychosis and off‐label use to manage insomnia, delirium and dementia. Regular assessment of patients taking benzodiazepines and antipsychotic medications and their plans of care is essential for nurses caring for older adults.

## CONFLICT OF INTEREST

There are no financial conflicts of interest to disclose.

## AUTHORS CONTRIBUTIONS

Dr. Neft, Mrs. Oerther, Dr. Halloway, Dr. Hanneman and Dr. Mitchell contributed to writing this manuscript and revising the manuscript critically for important intellectual content.
